# Emergency Laparoscopic Cholecystectomy Pathway Reduces Elective Waiting Times and Preoperative Admissions: A Prospective Propensity-Matched Cohort Study

**DOI:** 10.3390/medsci13030086

**Published:** 2025-06-27

**Authors:** Mohammed Hamid, Omar E. S. Mostafa, Maria Kausar, Amina Amin, Oladapo Olajumoke, Abhinav Singhal, Gowtham Bharnala, Akinfemi Akingboye, Ricardo Camprodon, Chaminda Sellahewa

**Affiliations:** 1 General Surgery Department, Russell’s Hall Hospital, Dudley Group NHS Foundation Trust, Pensnett Rd, Dudley DY1 2HQ, UK; 2 Aston Medical School, College of Health and Life Science, Aston University, Birmingham B4 7ET, UK

**Keywords:** emergency cholecystectomy, waiting list, preoperative admissions, subtotal cholecystectomy, postoperative ERCP, patient outcomes, service cost

## Abstract

Background: Emergency laparoscopic cholecystectomy (ELC) has emerged as a viable alternative to delayed elective surgery for acute gallstone disease, although its widespread adoption is hindered by cultural barriers. This study compares outcomes between elective and emergency laparoscopic cholecystectomy and evaluates the impact of implementing an ELC pathway on elective waiting times, patient outcomes, and overall service delivery. Methods: A prospective cohort study was conducted between December 2021 and December 2023, including all patients undergoing emergency or elective laparoscopic cholecystectomy. One-to-one propensity score matching, correlation statistics, and multivariate logistic regression were used to analyse outcomes. Results: Of 585 patients, 314 (53.4%) underwent emergency and 271 (46.3%) elective cholecystectomies. After matching, 474 patients were analysed (237 per group). The ELC pathway achieved an 81.4% first-presentation procedure rate, with 69.2% managed as day cases and 84.4% discharged the following day. Emergency cases had longer operative times (+9 min), higher rates of subtotal cholecystectomy (8.9% vs. 3.0%, *p* < 0.001), and more frequent postoperative ERCP (16.9% vs. 4.6%, *p* < 0.001). Other outcomes were comparable. Introduction of the ELC pathway significantly reduced elective waiting times from a median of nine to three months (R = −0.219, R^2^ = 0.059, *p* < 0.001) and preoperative admissions (IQR 0–1, R = −0.223, R^2^ = 0.050, *p* = 0.002). Conclusions: An ELC pathway is a safe and effective alternative to elective gallstone surgery, offering substantial benefits to patients and healthcare systems, while serving as a strategic, cost-conscious approach to reducing surgical waiting times and preoperative admissions. Its success hinges upon surgical expertise in acute decision making, skill in performing subtotal cholecystectomy, and access to institutional resources such as advanced imaging and ERCP services.

## 1. Introduction

Surgical waiting times have become a critical global healthcare issue, exacerbated by the COVID-19 pandemic, which significantly disrupted elective surgical services and led to widespread procedural backlogs [1,2,3]. In the United Kingdom (U.K.) alone, by mid-2023, over 7.6 million people were reported to be on elective surgical waiting lists, with similar patterns observed internationally [1,4]. Gallstone disease is one of the leading causes of surgical admissions, and delays in elective laparoscopic cholecystectomy are associated with increased rates of emergency readmissions, complications, and unplanned surgery [5,6,7].

In response, emergency laparoscopic cholecystectomy (ELC) pathways, often referred to as “hot gallbladder” pathways, are increasingly being adopted, particularly for the urgent management of symptomatic cholelithiasis, acute cholecystitis, and gallstone pancreatitis [8,9]. Evidence supports the safety and efficacy of ELC, demonstrating comparable perioperative outcomes to delayed surgery, while offering additional benefits such as reduced hospital stays, lower readmission rates, cost-effectiveness, faster return to normal activity, and improved patient-reported outcomes [9,10,11,12,13].

Despite these advantages, the transition from a culture of delayed elective surgery to routine acceptance of emergency pathways remains a challenge, often hindered by institutional inertia and long-standing clinical paradigms. This study aims to compare outcomes between emergency and elective laparoscopic cholecystectomy, as well as to evaluate the broader impact of introducing an emergency cholecystectomy pathway on elective waiting times, patient outcomes, and service delivery.

## 2. Methods

### 2.1. Study Design and Setting

A prospective cohort study was conducted to explore patient outcomes and service delivery for elective and emergency cholecystectomy cases between December 2021 and December 2023. The study was undertaken at a district general hospital in the United Kingdom. Institutional approval was obtained prior to data collection (registration number: GENSUR/SE/2021-22/35). The study was conducted in accordance with the Declaration of Helsinki and reported in line with the STROCSS 2024 guidelines.

**Figure 1:**
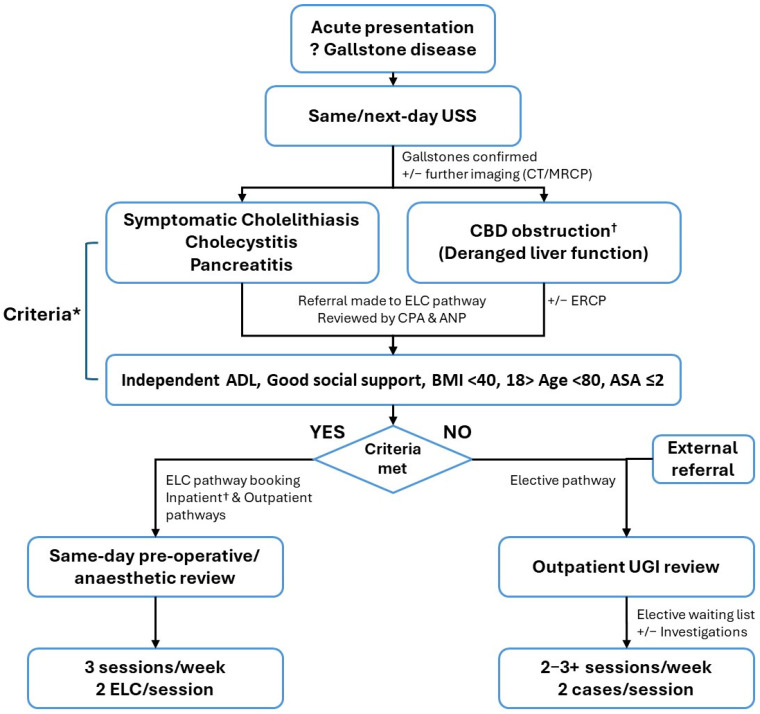
The local cholecystectomy service. Emergency laparoscopic cholecystectomy (ELC) sessions were made possible by reorganising elective activities. Procedures were primarily undertaken by experienced upper gastrointestinal (UGI) surgeons and their trainees, with general surgeons covering on the odd planned occasion. * On occasion, patients falling outside these criteria (e.g., ASA 3+) were pragmatically enrolled onto the ELC pathway on clinical grounds and when an experienced UGI surgeon was available. ^†^ Patients with an obstructive picture were often booked onto the inpatient ELC pathway once appropriate investigations were complete. USS: ultrasound, CT: computed tomography scan, MRCP: magnetic resonance cholangiopancreatography, CBD: common bile duct, ERCP: endoscopic retrograde cholangiopancreatography, CPA: clinical physician associate, ANP: advanced nurse practitioner, ADL: activities of daily living, BMI: body mass index, ASA: American Society of Anaesthesiologists.

At the start of the study, the Trust implemented an ELC pathway to facilitate a shift in practice and culture. The local cholecystectomy service incorporating the novel ELC pathway is outlined in Figure 1.

### 2.2. Patient Selection Criteria

The selection criteria for the ELC pathway are summarised in Figure 1. Patients presenting acutely with a clinical and radiological diagnosis of symptomatic cholelithiasis, calculous cholecystitis, or gallstone pancreatitis, as well as those with successful clearance of common bile duct obstruction via ERCP, are eligible for ELC if they meet the following criteria: independent activities of daily living, adequate social support, BMI < 40, age between 18 and 80 years, and ASA score ≤ 2. On occasion, patients outside these criteria (e.g., ASA 3+) were pragmatically included in the ELC pathway based on clinical judgment and availability of an experienced upper gastrointestinal surgeon. Cases not meeting the ELC criteria were referred to an upper gastrointestinal (UGI) surgeon for assessment and management in the elective setting. Any patient aged over 18 years with gallstone disease requiring cholecystectomy and deemed suitable for general anaesthesia was eligible for inclusion in the elective surgery pathway. Patients were also referred to the elective pathway via external routes, such as community practitioners.

### 2.3. Data Collection

All patients undergoing cholecystectomy during the study period were recorded in a departmental database, and their surgical journeys were analysed. Eighteen patients were excluded due to duplication or insufficient data. Preoperative, operative, and postoperative data were extracted from electronic and physical patient records for eligible patients, with all data stored on an encrypted, password-protected computer. Collected data included demographics (age, sex, BMI, ASA grade), preoperative details (clinico-radiological diagnosis, Tokyo guideline (TG) grade, procedures performed, first presentation status, surgical delay, suitability for the outpatient pathway, preoperative readmissions), operative details (emergency or elective, laparoscopic or open conversion, subtotal cholecystectomy rate, operative cholangiogram, antibiotics, and drain numbers), and 30-day postoperative details (histology report, length of stay, readmissions (90-day), complications, and Clavien–Dindo (CD) grade classification for complications) [14,15]. Subtotal cholecystectomy is a bail-out technique used during difficult cholecystectomy when the critical view of safety cannot be achieved [16]. It involves partial gallbladder removal and is classified as either fenestrating (open remnant) or reconstituting (closed remnant) [17]. Data were scrutinised by at least two study investigators, and predefined outcomes were extracted for all patients.

### 2.4. Outcomes

The primary outcome of interest was the impact of introducing an ELC pathway on elective waiting times, readmissions (pre- and postoperative), and procedural outcomes (complications and length of stay). Secondary outcomes included the comparison of postoperative outcomes between emergency and elective cholecystectomy.

### 2.5. Data Analysis

Two propensity score-matched (PSM) groups of near-equal size for elective versus ELC cases were generated using one-on-one near-neighbour matching with a caliper of 0.1. Matching variables included age, gender, BMI, and ASA grade. A standardised mean difference (SMD) of less than 0.1 was considered negligible, indicating appropriate matching (Supplementary Figure S1).

The Kolmogorov–Smirnov test was used to assess normality, with a *p*-value of less than 0.05 considered significant. Matched and unmatched data are summarised using median and interquartile range (IQR) for continuous variables and number and percentage for categorical data. Comparisons between emergency and elective groups were performed using Mann–Whitney U tests for continuous variables and Chi-squared tests for categorical variables. The odds ratio (OR) and 95% confidence interval (95% CI) were calculated for patient factors and outcomes associated with both primary and secondary outcomes using univariate and multivariate binomial logistic regression (with a priori covariates: age, sex, ASA, and BMI). Correlation statistics (Pearson correlation coefficient (R) and coefficient of determination (R^2^)) were used to analyse primary outcomes. *p*-values of <0.05 were considered statistically significant. Statistical analysis was performed using GraphPad Prism V10.4.2 (GraphPad Software, LLC.) and R V4.3.2 (R Foundation for Statistical Computing, Vienna, Austria).

## 3. Results

### 3.1. Study Patient Characteristics

A total of 585 unmatched patients were included, of whom 314 (53.4%) underwent emergency cholecystectomy and 271 (46.3%) underwent elective procedures. The median age was 52 years (IQR: 37–64), with 429 (73.3%) patients being female, 322 (55.0%) obese, and 135 (23.1%) classified as ASA grade 3+. Unmatched and matched (*n* = 474, 1:1, 237 elective, 237 emergency) patient characteristics are summarised in Table 1. Salient outcomes associated with preoperative patient characteristics are highlighted in Table 2.

Given the selective criteria for the emergency cholecystectomy pathway, patients were more likely to be postponed for an elective procedure if they were older (*p* < 0.001), had significant comorbidities (28%, *p* = 0.008), presented with symptomatic cholelithiasis or CBD obstruction (*p* < 0.001), or required further preoperative investigations (e.g., MRCP, *p* < 0.001; CT, *p* = 0.004, Table 1, unmatched). The emergency pathway favoured patients with a brief history of acute cholecystitis (*p* < 0.001), leading to more patients undergoing cholecystectomy on their first presentation (81.2–81.4%, *p* < 0.001), significantly reducing the need for additional preoperative readmissions (*p* < 0.001) (Table 1). These preoperative associations were consistent after matching for age, gender, BMI, and ASA (Table 1).

Elective procedures had a median waiting time of 232 days (231 days post-matching), compared to 5 days for emergency cholecystectomy. Emergency surgeries took an average of 9 min longer (median 69 vs. 60 min, *p* < 0.001), with patients more likely to require subtotal cholecystectomy (8.9%, *p* = 0.003) and postoperative ERCP (4.4–4.6%, *p* < 0.001) regardless of matching. However, leak rates and iatrogenic common bile duct (CBD) injuries were similar in both cohorts. Table 3 and Table 4 present key patient factors and outcomes associated with subtotal cholecystectomy and postoperative ERCP, respectively. Elective patients were more likely to receive intraoperative antibiotics because most emergency cholecystectomy patients had received perioperative antibiotics in the ward or at home.

Other postoperative outcomes were comparable. Elective patients reported more postoperative pain (8.9%, *p* = 0.007), while emergency patients had higher rates of surgical site infections (SSI) (3.2%, *p* = 0.012) in the unmatched data. These differences became statistically insignificant after matching, suggesting that the observed disparity was due to baseline differences between the groups (e.g., male gender, obesity) rather than the timing of surgery itself [18]. In the unmatched data (Table 1), the emergency surgery group included 3% more male patients and had a slightly higher BMI interquartile range (IQR 28–36 vs. 28–35), which may have contributed to the increased SSI rate observed. Other patient-level risk factors identified in the literature (e.g., diabetes, smoking-related diseases, chronic anaemia, malnutrition, and drug abuse) were not specifically assessed in our dataset, and therefore their impact prior to matching could not be evaluated [18,19]. Day-case rates were consistent (median 70.9–71.3%), although elective patients were more likely to go home the next day if an overnight stay was required (93.7%, *p* = 0.002). There were no significant differences in overall length of stay, high-grade complications, or mortality rates between the elective and emergency groups.

### 3.2. Impact of the ELC Pathway on Services

**Figure 2:**
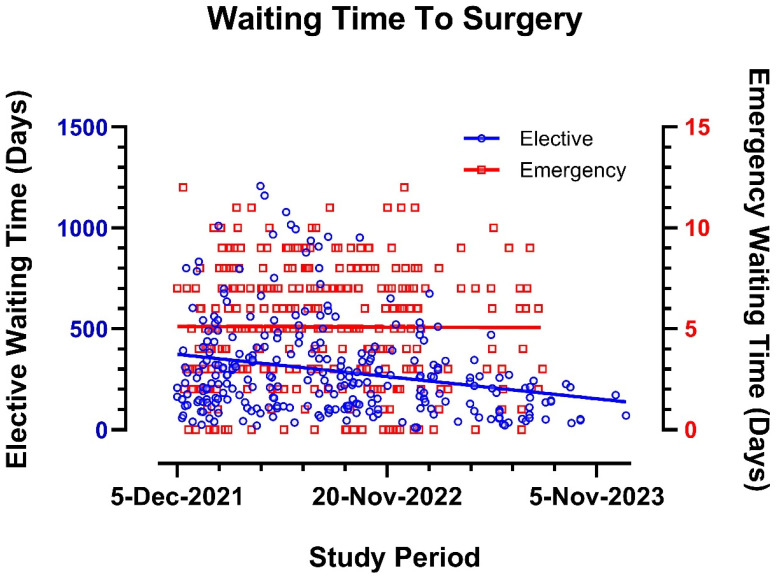
Waiting time for elective and emergency surgery during the study period.

**Figure 3:**
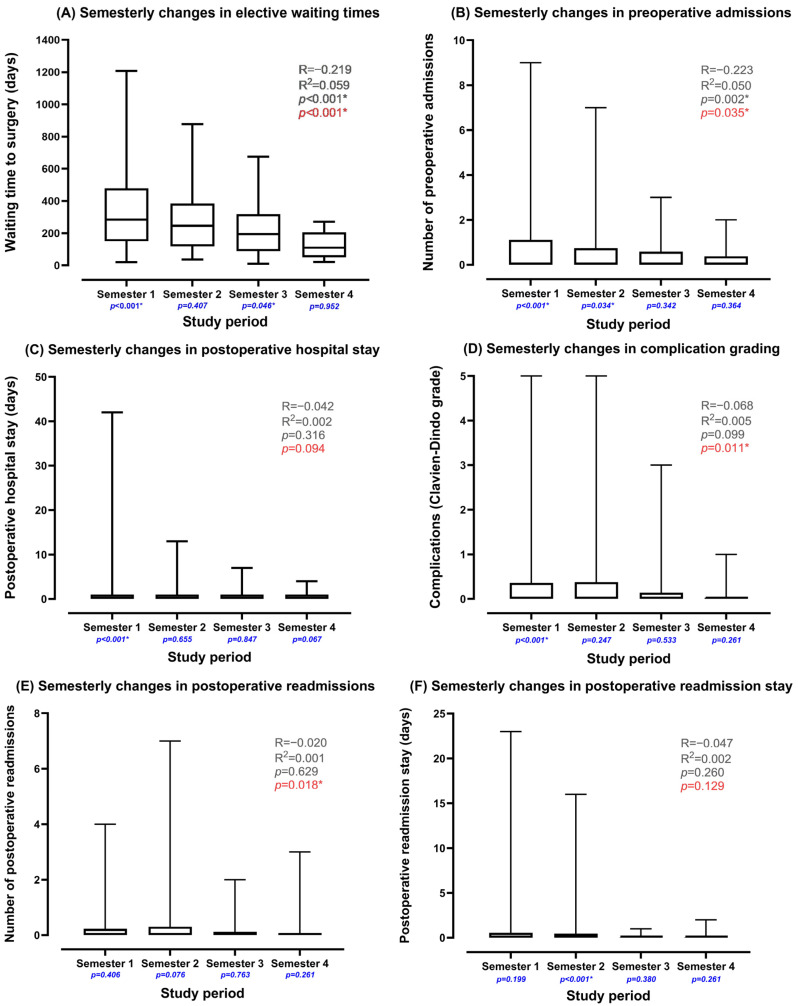
Effect of establishing an emergency laparoscopic cholecystectomy pathway on the local cholecystectomy service. * Indicates statistically significant. Unmatched data. *p*-values associated with changes per semester (6-month periods) are highlighted in blue; red *p*-values relate to comparison between the first and last semester; and grey statistics correspond to the entire study period. R: Pearson correlation coefficient, R^2^: coefficient of determination. The number of samples in each semester: (**A**) (Semesters (*n*)–1 (101), 2 (86), 3 (42), 4 (42)); (**B**–**F**) (Semesters (*n*)–1 (211), 2 (230), 3 (81), 4 (63)).

The introduction of the emergency pathway significantly reduced elective surgery waiting times from a median of 284 days (~9 months) in the first study semester to 110 days (~3 months) in the final semester (R = −0.219, R^2^ = 0.059, *p* < 0.001, Figure 2 and Figure 3A). This was coupled with an increased rate of first-presentation surgeries (81.2–81.4%, *p* < 0.001). Preoperative admissions were significantly reduced throughout the study period (R = −0.223, R^2^ = 0.050, *p* = 0.002, Figure 3B), with substantial improvements in the first and second semesters (*p* < 0.001 and *p* = 0.034, respectively), although the significance diminished in later semesters. Postoperative hospital stay was significantly reduced in the first semester (*p* < 0.001), with a non-significant reduction across the full study period (Figure 3C). There was a significant reduction in postoperative complication grading and readmissions between the first and final semesters (*p* = 0.011 and *p* = 0.018, Figure 3D,E, respectively), although these correlations were not significant when considering the entire study period. Total readmission stays were significantly reduced in the second semester (*p* < 0.001), though the reduction was not statistically significant across the full study period (Figure 3F).

## 4. Discussion

This study highlights the safety profile and efficacy of the emergency laparoscopic cholecystectomy (ELC) paradigm compared to the delayed elective surgery model, demonstrating similar postoperative outcomes, the absence of deleterious consequences, and additional benefits for both patients and healthcare services. While postoperative endoscopic retrograde cholangiopancreatography (ERCP) was performed more frequently in the emergency cohort, the incidence of leaks or retained stones did not differ significantly between the groups. Surgeons managing “hot gallbladders” should consider subtotal cholecystectomy as an essential technique to minimise the risk of common bile duct (CBD) injuries.

The introduction of an ELC pathway has significant advantages in gallbladder disease management, including immediate reductions in elective waiting times and preoperative readmissions, both of which are correlated with improved postoperative outcomes and reduced hospital costs. Additionally, the ELC pathway facilitated offering surgery to a greater number of patients at first presentation, indirectly improving overall postoperative length of stay, complication rates, and readmission rates during the study period.

Numerous randomised controlled trials (RCTs) comparing early versus delayed laparoscopic cholecystectomy for acute cholecystitis have been published, spanning over two decades. Meta-analyses of these studies consistently demonstrate that ELC is safe, shortens hospital stays, reduces hospital costs, decreases workdays lost, and improves patient satisfaction when compared to delayed cholecystectomy [20,21]. Similarly, outcomes for patients with symptomatic cholelithiasis and gallstone pancreatitis have demonstrated the efficacy of early intervention [10,22,23,24]. Although our study did not examine occupational outcomes or quality of life, it aligns with the findings of previous studies by showing benefits in terms of reducing complications and promoting quicker recovery.

Bourgouin et al. add an important dimension to the decision-making process and outcome prediction for operative timing by incorporating anticipated surgical difficulty, assessed using their own previously developed Difficult Laparoscopic Cholecystectomy (DiLC) score [25]. Their analysis demonstrates that patients with a DiLC score ≥ 10, indicating a technically challenging procedure, experience better outcomes when surgery is delayed. Conversely, early cholecystectomy in these high-risk cases was associated with a significantly increased risk of serious intraoperative and postoperative complications on multivariate analysis [26].

In our study, the number of subtotal cholecystectomies performed in the emergency cohort was significantly higher (8.9% vs. 3.0%, *p* < 0.001), though this falls within the expected range for national ELC practices, which is reported to be between 7% and 10% [27]. A subtotal cholecystectomy should not be regarded as a complication but rather as a valuable strategy to minimise risks such as CBD injury, conversions to open surgery, and other complications [28]. A recent systematic review and meta-analysis comparing subtotal versus total cholecystectomy for difficult gallbladders revealed that the subtotal approach significantly reduces the risk of CBD injury [29].

Furthermore, although postoperative ERCP was performed significantly more frequently in the emergency cohort (16.9% vs. 4.6%, *p* < 0.001), the incidence of leaks or retained stones was not significantly higher. Postoperative ERCP was significantly associated with factors such as preoperative ERCP (OR 43.84, *p* < 0.001), delayed surgery (>1 year, OR 3.66, *p* = 0.036), multiple preoperative readmissions (OR 7.64, *p* = 0.001), and intraoperative subtotal cholecystectomy (OR 3.19, *p* = 0.016), as outlined in Table 4.

Prolonged waiting times for surgery increase the likelihood of recurrent episodes of gallstone complications, which can result in localised changes such as fibrosis and distorted anatomy, leading to “difficult” cholecystectomies. These challenges increase the rate of intra- and postoperative complications and morbidity [26,30]. Long waiting times also raise the probability of multiple hospital admissions, further escalating the need for emergency surgery [31,32,33]. Our study demonstrates how the introduction of an ELC pathway not only provides a safe and effective approach for managing acute gallstone disease but also significantly reduces elective waiting times and preoperative readmissions, while improving overall hospital stays, complication grading, and readmission rates over a two-year period.

Other strategies to address the prolonged waiting times for elective cholecystectomy have emerged since the COVID-19 pandemic, including the use of private sector services, opening additional surgical sites, mobile theatres, and weekend surgery initiatives [9,34]. However, these approaches often lead to increased operational costs. In contrast, the implementation of an ELC pathway offers a cost-effective strategy to reduce elective waiting times by increasing the rate of first-presentation procedures, reducing preoperative admissions, and mitigating the associated complications and readmission costs. Early cholecystectomy has been shown to cost approximately GBP 150 less than delayed surgery. While this may seem negligible per individual case, the cumulative savings could amount to GBP 3.8 million annually across the U.K. [35]. Macafee et al. also demonstrated that while the cost of early surgery is only marginally lower, the incremental cost per quality-adjusted life year (QALY) favours elective management [36]. Despite this, no study has yet fully examined the long-term fiscal impact of implementing an ELC pathway on elective waiting lists, which could be transformative in an era of cost containment.

The greatest challenge to widespread adoption of ELC, both in the U.K. and globally, lies in the cultural shift required within surgical departments. Engagement at all levels is crucial for success [12,32,37]. Locally, within two years of introducing the ELC pathway, the proportion of patients undergoing surgery at first presentation increased from 5% to over 80%. Emergency surgery waiting times dropped from 67 days to just 5, while elective surgery waiting times were reduced from over 9 months to under 3 months. Notably, more than half of all cholecystectomies were performed as “hot” procedures [9].

## 5. Limitations

This study is observational in nature and is limited by its non-randomised design. Differences in patient demographics between the two groups, due to the selection criteria for the emergency pathway, were addressed through propensity score matching. However, the results are specific to a single institution, and the findings may not be generalisable to other regions or healthcare systems. Additionally, all procedures were primarily performed by advanced upper gastrointestinal surgeons and their trainees, which may have implications for the generalisability of the outcomes. Local cost-utility analysis was not included in this study and remains under evaluation.

## 6. Conclusions

The emergency laparoscopic cholecystectomy (ELC) pathway is a safe and effective alternative to delayed elective surgery, offering substantial benefits to both patients and healthcare services. This approach significantly reduces elective waiting times and preoperative admissions, which are known to negatively impact patient outcomes. Moreover, the ELC pathway increases the rate of first-presentation procedures and improves overall postoperative outcomes, including reduced hospital stays, complications, and readmissions. The financial implications of implementing an ELC pathway are considerable, with potential cost savings at both the local and national levels. As “hot gallbladders” often require subtotal cholecystectomy, this technique should be an integral part of the surgical skill set for those performing emergency cholecystectomy. Widespread adoption of ELC will require a cultural shift within surgical departments, but its benefits in terms of patient care, service delivery, and cost-effectiveness make it a compelling model for the future management of gallstone disease.

## Figures and Tables

**Table 1:** Matched and unmatched study patient characteristics, each compared between elective and emergency cholecystectomy cases.

Characteristic	Unmatched				Matched			
	All (*N* = 585)	Emergency (*n* = 314)	Elective (*n* = 271)	*p*-Value	All (*N* = 474)	Emergency (*n* = 237)	Elective (*n* = 237)	*p*-Value
Age, median (IQR), years	52 (37–64)	48 (33–60)	55 (41–68)	<0.001 ^†^	52 (38–64)	52 (38–63)	52 (39–64)	0.561
Female sex, *n* (%)	429 (73.3)	224 (71.3)	205 (75.7)	0.240	352 (74.3)	176 (74.3)	176 (74.3)	>0.999
BMI, median (IQR)	31 (27–36)	32 (28–36)	31 (27–36)	0.120	32 (27–36)	32 (28–35)	31 (27–36)	0.573
BMI 30–34.9; Class I obesity	159 (27.2)	87 (27.7)	72 (26.6)	0.758	123 (25.9)	62 (26.2)	61 (25.7)	>0.999
BMI 35–39.9; Class II obesity	106 (18.1)	60 (19.1)	46 (17.0)	0.504	87 (18.4)	43 (18.1)	44 (18.6)	>0.999
BMI 40+; Class III obesity	57 (9.7)	24 (7.6)	33 (12.2)	0.065	50 (10.5)	19 (8.0)	31 (13.1)	0.099
ASA Grade 3+, *n* (%)	135 (23.1)	59 (18.8)	76 (28.0)	0.008 *	102 (21.5)	45 (19.0)	57 (24.1)	0.219
Imaging and clinical diagnosis, *n* (%) ^								
Symptomatic cholelithiasis	361 (61.7)	108 (34.4)	253 (93.4)	<0.001 *	305 (64.3)	83 (35.0)	222 (93.7)	<0.001 *
Cholecystitis	286 (48.9)	176 (56.1)	110 (40.6)	<0.001 *	228 (48.1)	133 (56.1)	95 (40.1)	<0.001 *
CBD obstruction	99 (16.9)	29 (9.2)	70 (25.8)	<0.001 *	79 (16.7)	23 (9.7)	56 (23.6)	<0.001 *
Cholangitis	44 (7.5)	12 (3.8)	32 (11.8)	<0.001 *	39 (8.2)	10 (4.2)	29 (12.2)	0.002 *
Pancreatitis	76 (13.0)	46 (14.7)	30 (11.1)	0.199	60 (12.7)	34 (14.3)	26 (11.0)	0.334
Imaging modality used, *n* (%) ^								
USS	528 (90.3)	285 (90.8)	243 (89.7)	0.656	422 (89.0)	210 (88.6)	212 (89.5)	0.883
MRCP	103 (17.6)	38 (12.1)	65 (24.0)	<0.001 *	85 (17.9)	33 (13.9)	52 (21.9)	0.031 *
ERCP	87 (14.9)	49 (15.6)	38 (14.0)	0.592	72 (15.2)	40 (16.9)	32 (13.5)	0.371
Pre-op	68 (11.6)	39 (12.4)	29 (10.7)	0.518	54 (11.4)	30 (12.7)	24 (10.1)	0.470
Post-op	61 (10.4)	49 (15.6)	12 (4.4)	<0.001 *	51 (10.8)	40 (16.9)	11 (4.6)	<0.001 *
CT	113 (19.3)	47 (15.0)	66 (24.4)	0.004 *	93 (19.6)	39 (16.5)	54 (22.8)	<0.001 *
First presentation, *n* (%)	385 (65.8)	255 (81.2)	130 (48.0)	<0.001 *	302 (63.7)	193 (81.4)	109 (46.0)	<0.001 *
Multiple admissions (IQR)	0 (0–1)	0 (0–0)	1 (0–1)	<0.001 ^†^	0 (0–1)	0 (0–0)	1 (0–1)	<0.001 ^†^
Surgery WT (IQR), days	9 (5–217)	5 (3–7)	232 (118–381)	<0.001 ^†^	12 (5–231)	5 (3–7)	231 (119–387)	<0.001 ^†^
SDEC outpatient, *n* (%)	-	241 (76.8)	-	-	-	183 (58.2)	-	-
Operative details, *n* (%)								
Laparoscopic	584 (99.8)	313 (99.7)	271 (100.0)	0.353	473 (99.8)	236 (99.6)	237 (100.0)	>0.999
Operative duration	64 (53–81)	69 (58–88)	60 (49–73)	<0.001 ^†^	64 (52–80)	69 (57–88)	60 (49–73)	<0.001 *
Subtotal cholecystectomy	36 (6.2)	28 (8.9)	8 (3.0)	0.003 *	28 (5.9)	21 (8.9)	7 (3.0)	0.010 *
Fenestrated	11 (1.9)	9 (2.9)	2 (0.7)	0.059	23 (4.9)	21 (8.9)	2 (0.8)	<0.001 *
Reconstructing	25 (4.3)	19 (6.1)	6 (2.2)	0.022 *	5 (1.1)	0 (0.0)	5 (2.1)	0.061
On-table cholangiogram	202 (34.5)	118 (37.6)	84 (31.0)	0.095	155 (32.7)	86 (36.3)	69 (29.1)	0.117
Drain insertion	85 (14.5)	51 (16.2)	34 (12.6)	0.206	72 (15.2)	41 (17.3)	31 (13.1)	0.798
Antibiotics intra-operatively	242 (41.4)	68 (21.7)	174 (64.2)	<0.001 *	204 (43.0)	53 (22.4)	151 (63.7)	<0.001 *
Histology report, *n* (%)								
Acute inflammation	99 (16.9)	85 (27.1)	14 (5.1)	<0.001 *	79 (16.7)	68 (28.7)	11 (4.6)	<0.001 *
Chronic inflammation	485 (82.9)	229 (72.9)	256 (93.4)	<0.001 *	395 (83.3)	169 (71.3)	226 (95.4)	<0.001 *
Adenocarcinoma	1 (0.2)	0 (0.0)	1 (0.4)	0.281	0 (0.0)	0 (0.0)	0 (0.0)	-
30-day outcomes								
Complications, *n* (%)	89 (15.2)	48 (15.3)	41 (15.1)	0.958	74 (15.6)	37 (15.6)	37 (15.6)	>0.999
Collection	11 (1.9)	6 (1.9)	5 (1.9)	0.953	10 (2.1)	5 (2.1)	5 (2.1)	>0.999
Hernia	2 (0.3)	1 (0.3)	1 (0.4)	0.917	2 (0.4)	1 (0.4)	1 (0.4)	>0.999
Bile leak	11 (1.9)	7 (2.2)	4 (1.5)	0.504	10 (2.1)	6 (2.5)	4 (1.7)	0.751
Pain	35 (6.0)	11 (3.5)	24 (8.9)	0.007 *	31 (6.5)	11 (4.6)	20 (8.4)	0.136
Pancreatitis	4 (0.7)	2 (0.6)	2 (0.7)	0.882	4 (0.8)	2 (0.8)	2 (0.8)	>0.999
Retained stone	8 (1.4)	5 (1.6)	3 (1.1)	0.614	7 (1.5)	4 (1.7)	3 (1.3)	>0.999
Sepsis	2 (0.3)	2 (0.6)	0 (0.0)	0.188	2 (0.4)	2 (0.8)	0 (0.0)	0.499
Shortness of breath	2 (0.3)	2 (0.6)	0 (0.0)	0.188	2 (0.4)	2 (0.8)	0 (0.0)	0.499
SSI	11 (1.9)	10 (3.2)	1 (0.4)	0.012 *	4 (0.8)	3 (1.3)	1 (0.4)	0.623
Early return to theatre	1 (0.2)	1 (0.3)	0 (0.0)	0.353	0 (0.0)	0 (0.0)	0 (0.0)	-
Clavien–Dindo grade 3+, *n* (%)	36 (6.2)	24 (7.6)	12 (4.4)	0.107	30 (6.3)	19 (8.0)	11 (4.6)	0.186
Length of stay (IQR), days	0 (0–1)	0 (0–1)	0 (0–1)	0.336	0 (0–1)	0 (0–1)	0 (0–1)	0.110
Day case, *n* (%)	415 (70.9)	221 (70.4)	194 (71.6)	0.749	338 (71.3)	164 (69.2)	174 (73.4)	0.361
Next-day discharge, *n* (%)	523 (89.4)	269 (85.7)	254 (93.7)	0.002 *	422 (89.0)	200 (84.4)	222 (93.7)	0.002 *
Mortality, *n* (%)	2 (0.3)	1 (0.3)	1 (0.4)	0.917	2 (0.4)	1 (0.4)	1 (0.4)	>0.999
90-day post-op reattendance, *n* (%)	94 (16.1)	53 (16.9)	41 (15.1)	0.566	78 (16.5)	41 (17.3)	37 (15.6)	0.710
Planned	26 (4.4)	15 (4.8)	11 (4.1)	0.674	21 (4.4)	12 (5.1)	9 (3.8)	0.656
Emergency	68 (11.6)	38 (12.1)	30 (11.1)	0.698	57 (12.0)	29 (12.2)	28 (11.8)	>0.999
Readmission	40 (6.8)	25 (8.0)	15 (5.5)	0.246	58 (12.2)	27 (11.3)	31 (13.1)	0.674
Multiple reattendances	21 (3.6)	15 (4.8)	6 (2.2)	0.097	20 (4.2)	14 (4.6)	6 (2.5)	0.108
Additional LOS (IQR), days	5 (3–8)	6 (4–9)	5 (2–6)	0.237	5 (3–8)	6 (5–9)	5 (2–8)	0.255

^ Indicates multifactorial component * Indicates statistically significant using Chi-squared analysis. ^†^ Indicates statistically significant using Mann–Whitney U test. IQR: interquartile range, BMI: body mass index, ASA: American Society of Anaesthesiologists, CBD: common bile duct, USS: ultrasound scan, MRCP: magnetic resonance cholangiopancreatography, ERCP: endoscopic retrograde cholangiopancreatography, CT: computerised tomography scan, WT: waiting time, SDEC: same-day emergency care pathway, Pre-op: preoperatively, Post-op: postoperatively, SSI: surgical site infection, LOS: length of stay.

**Table 2:** Preoperative characteristics and associated outcomes using univariate and multivariate binomial logistic regression models.

Characteristic	Univariate OR (95% CI)	*p*-Value	Multivariate OR (95% CI)	*p*-Value
Age < 40 years				
Drain insertion	0.34 (0.17, 0.68)	0.004	0.36 (0.18, 0.72)	0.002
Antibiotics	0.55 (0.37, 0.81)	0.011	0.59 (0.40, 0.89)	0.011
Reattendance via ED	12.29 (1.55, 97.24)	0.009	16.47 (1.99, 136.41)	<0.001
Age 40–60 years				
Reattendance via ED	0.49 (0.18, 1.30)	0.080 ^§^	0.40 (0.14, 1.12)	0.078 ^§^
Age > 60 years				
Drain insertion	2.03 (1.23, 3.35)	0.012	1.96 (1.16, 3.30)	0.013
Antibiotics	1.95 (1.34, 2.83)	0.002	1.82 (1.24, 2.68)	0.002
Day case procedure	0.57 (0.38, 0.85)	0.055 ^§^	0.66 (0.43, 1.01)	0.056 ^§^
Female gender				
Subtotal cholecystectomy	0.46 (0.22, 0.95)	0.030	0.44 (0.21, 0.93)	0.034
ASA 1				
Histology: Acute Inf’	2.15 (0.96, 4.81)	0.062 ^§^	2.30 (0.96, 5.52)	0.073 ^§^
ASA 2				
Antibiotics	0.59 (0.41, 0.87)	0.019	0.63 (0.43, 0.93)	0.019
Day case procedure	2.29 (1.53, 3.42)	<0.001	2.13 (1.40, 3.23)	<0.001
Next day discharge	0.46 (0.29, 0.72)	0.003	0.48 (0.30, 0.78)	0.003
ASA 3				
Antibiotics	1.84 (1.23, 2.76)	0.031	1.61 (1.04, 2.50)	0.031
Day case procedure	0.34 (0.22, 0.52)	<0.001	0.38 (0.24, 0.60)	<0.001
Next day discharge	2.87 (1.79, 4.60)	<0.001	2.69 (1.61, 4.50)	<0.001
Two-day stay	2.62 (1.08, 6.38)	0.052 ^§^	2.56 (0.99, 6.59)	0.059 ^§^
BMI 30–35				
On-table cholangiogram	0.65 (0.43, 0.98)	0.039	0.65 (0.43, 0.98)	0.036
Prolonged hospital stays	2.25 (0.93, 5.40)	0.055 ^§^	2.39 (0.98, 5.81)	0.059 ^§^
Clavien–Dindo 3	0.35 (0.12, 1.02)	0.063 ^§^	0.36 (0.12, 1.05)	0.038
BMI > 35–40				
Day case procedure	1.84 (1.08, 3.16)	0.034	1.82 (1.05, 3.17)	0.028
Next-day discharge	0.53 (0.28, 1.01)	0.064 ^§^	0.54 (0.28, 1.04)	0.051 ^§^
Multiple post-op admissions	1.98 (1.18, 3.32)	0.007	2.07 (1.22, 3.50)	0.008
Reattendance via ED	0.39 (0.14, 1.08)	0.060 ^§^	0.35 (0.12, 1.04)	0.059 ^§^
Reattendance planned				
Clavien–Dindo 0	0.53 (0.31, 0.90)	0.013	0.50 (0.29, 0.86)	0.015
Clavien–Dindo 1	2.88 (1.38, 6.00)	0.003	3.07 (1.46, 6.46)	0.005
BMI ≥ 40				
On-table cholangiogram	1.44 (0.82, 2.52)	0.030	2.13 (1.07, 4.24)	0.030
Prolonged additional stay	1.12 (1.02, 1.23)	0.056 ^§^	1.13 (1.00, 1.27)	0.066 ^§^
Clavien–Dindo 3	3.41 (1.44, 8.06)	0.046	3.11 (1.02, 9.49)	0.049

^§^ Indicates NOT statistically significant. 95% CI: 95% confidence interval, <: less than, >: greater than, ≥: greater than or equal to, ED: emergency department, ASA: American Society of Anaesthesiologists, Inf’: inflammation, BMI: body mass index, Post-op: postoperative.

**Table 3:** Significant factors and outcomes associated with subtotal cholecystectomy using univariate and multivariate binomial logistic regression modelling.

Characteristic	Univariate OR (95% CI)	*p*-Value	Multivariate OR (95% CI)	*p*-Value
Male gender	2.17 (1.06, 4.45)	0.030	2.28 (1.08, 4.79)	0.034
Biliary colic	0.18 (0.08, 0.39)	<0.001	0.18 (0.08, 0.39)	<0.001
Cholecystitis	6.54 (2.48, 17.2)	<0.001	6.40 (2.41, 16.98)	<0.001
Higher Tokyo grading	6.87 (2.95,16.03)	<0.001	6.37 (2.66, 15.25)	<0.001
Elective WT > 1 year	2.80 (0.68, 11.49)	0.027	2.29 (0.53, 9.87)	0.027
ELC OPD pathway	0.28 (0.11, 0.68)	0.012	0.30 (0.12, 0.77)	0.022
First presentation	0.39 (0.19, 0.79)	0.017	0.41 (0.20, 0.85)	0.017
Multiple pre-op admissions	1.37 (1.12, 1.69)	0.008	1.34 (1.08, 1.65)	0.013
1 admission	1.68 (0.77, 3.63)	0.209 ^§^	1.66 (0.75, 3.63)	0.222 ^§^
2–3 admissions	1.32 (0.49, 3.56)	0.733 ^§^	1.19 (0.44, 3.25)	0.737 ^§^
≥4 admissions	6.83 (2.02, 23.09)	0.005	5.91 (1.69, 20.73)	0.014
Drain insertion	28.44 (12.18, 66.39)	<0.001	31.62 (12.83, 77.94)	<0.001
Antibiotics	4.81 (2.13, 10.87)	<0.001	4.85 (2.09, 11.24)	<0.001
On-table cholangiogram	0.19 (0.06, 0.63)	0.006	0.19 (0.06, 0.62)	<0.001
Histology: Acute inf’	9.19 (4.39, 19.21)	<0.001	9.53 (4.43, 20.50)	<0.001
Histology: Chronic inf’	0.11 (0.05, 0.23)	<0.001	0.10 (0.05, 0.23)	<0.001
Day case procedure	0.10 (0.04, 0.22)	<0.001	0.09 (0.04, 0.22)	<0.001
Next day discharge	4.36 (2.11, 9.01)	<0.001	4.28 (2.01, 9.12)	<0.001
Prolonged hospital stays	1.13 (0.99, 1.29)	0.053	1.13 (1.00, 1.27)	0.022
2-day stay	3.96 (1.25, 12.53)	0.022	3.98 (1.22, 12.98)	0.041
≥1-week stay	32.52 (2.87, 368.5)	0.005	37.5 (3.03, 463.66)	0.005
Postoperative readmission	4.36 (2.09, 9.08)	<0.001	3.97 (1.88, 8.37)	<0.001
Attendance via ED	0.18 (0.05, 0.60)	0.011	0.19 (0.05, 0.68)	0.010
Attendance planned	5.62 (1.68, 18.81)	0.011	5.39 (1.46, 19.87)	0.010
Multiple post-op admissions	1.60 (1.14, 2.24)	0.022	1.49 (1.06, 2.10)	0.034
Postoperative ERCP	3.35 (1.43, 7.88)	0.010	3.15 (1.31, 7.59)	0.018
Clavien–Dindo 0	0.24 (0.12, 0.51)	<0.001	0.28 (0.13, 0.59)	0.002
Clavien–Dindo 3	4.45 (1.68, 11.81)	0.009	3.80 (1.39, 10.38)	0.018

^§^ Indicates NOT statistically significant. 95% CI: 95% confidence interval, WT: waiting time, <: less than, >: greater than, ≥: greater than or equal to, Pre-op: preoperative, Inf’: inflammation, ED: emergency department, Post-op: postoperative, ERCP: endoscopic retrograde cholangiopancreatography.

**Table 4:** Significant factors and outcomes associated with postoperative ERCP using univariate and multivariate binomial logistic regression modelling.

Characteristic	Univariate OR (95% CI)	*p*-Value	Multivariate OR (95% CI)	*p*-Value
CBD obstruction	6.25 (3.43, 11.41)	<0.001	6.50 (3.50, 12.08)	<0.001
Cholangitis	4.53 (2.24, 9.97)	<0.001	4.57 (2.14, 9.77)	<0.001
Preoperative MRCP	2.54 (1.36, 4.76)	0.004	2.56 (1.35, 4.87)	0.006
Preoperative ERCP	36.43 (18.06,73.45)	<0.001	43.84 (20.51,93.68)	<0.001
Elective WT >1 year	4.07 (1.25, 13.27)	0.036	3.66 (1.09, 12.32)	0.036
ELC OPD pathway	0.05 (0.03, 0.10)	<0.001	0.05 (0.02, 0.10)	<0.001
First presentation	0.42 (0.23, 0.72)	0.003	0.42 (0.23, 0.75)	0.003
Multiple pre-op admissions	1.61 (1.33, 1.97)	<0.001	1.60 (1.31, 1.96)	<0.001
1 admission	0.87 (0.42, 1.80)	0.653 ^§^	0.85 (0.41, 1.75)	0.648 ^§^
2–3 admissions	2.78 (1.39, 5.55)	0.005	2.69 (1.34, 5.41)	0.009
≥4 admissions	7.79 (2.59, 23.43)	<0.001	7.64 (2.49, 23.38)	0.001
Subtotal cholecystectomy	3.35 (1.43, 7.88)	0.009	3.19 (1.33, 7.62)	0.016
Drain insertion	3.22 (1.68, 6.15)	0.001	3.04 (1.56, 5.93)	0.002
Antibiotics	2.45 (1.36, 4.41)	0.006	2.31 (1.27, 4.22)	0.005
Histology: Acute inf’	1.33 (0.64, 2.76)	0.476 ^§^	1.31 (0.62, 2.74)	0.485 ^§^
Histology: Chronic inf’	0.75 (0.36, 1.57)	0.476 ^§^	0.76 (0.36, 1.60)	0.485 ^§^
Day case procedures	0.24 (0.14, 0.44)	<0.001	0.25 (0.14, 0.46)	<0.001
Prolonged hospital stays	1.58 (1.31, 1.90)	<0.001	1.55 (1.29, 1.88)	<0.001
3–6-day stay	5.71 (2.29, 14.21)	<0.001	5.38 (2.14, 13.54)	0.001
≥1-week stay	19.40 (1.73, 217.74)	0.014	22.96 (1.87, 282.34)	0.014
Postoperative readmission	2.60 (1.37, 4.93)	0.004	2.57 (1.34, 4.91)	0.007
Prolonged additional stay	1.15 (1.05, 1.27)	0.004	1.15 (1.05, 1.27)	0.006
Clavien–Dindo 0	0.35 (0.19, 0.67)	0.002	0.36 (0.19, 0.69)	0.004
Clavien–Dindo 3	6.59 (2.94, 14.80)	<0.001	6.56 (2.87, 15.02)	<0.001

^§^ Indicates NOT statistically significant. 95% CI: 95% confidence interval, ERCP: endoscopic retrograde cholangiopancreatography, CBD: common bile duct, MRCP: magnetic resonance cholangiopancreatography, WT: waiting time, <: less than, >: greater than, ≥: greater than or equal to, ELC OPD: emergency laparoscopic cholecystectomy outpatient pathway, Pre-op: preoperative, Inf’: inflammation.

## Data Availability

Anonymised data will be made available upon request and only after institutional approval.

## Supplementary Materials

The following supporting information can be downloaded at: https://www.mdpi.com/article/10.3390/medsci13030086/s1, Figure S1: Propensity score matching graphs for age, gender, BMI and ASA—average standardized absolute mean difference (ASMD), Covariate balance, Histograms, Distribution of propensity scoring.
