# Supplementary material for: Emergency Laparoscopic Cholecystectomy Pathway Reduces Elective Waiting Times and Preoperative Admissions: A Prospective Propensity-Matched Cohort Study
Source: Med Sci (Basel). 2025 Jun 27;13(3):86. doi: 10.3390/medsci13030086 (PMC12286089; doi:10.3390/medsci13030086)
Supplement: Supplementary file 1 [file medsci-13-00086-s001.zip › medsci-3645738-supplementary.pdf]

Supplementary Figure S1: Propensity score matching graphs for age, gender, BMI and ASA - average standardized absolute mean difference (ASMD), Covariate balance, Histograms, Distribution of propensity scoring.

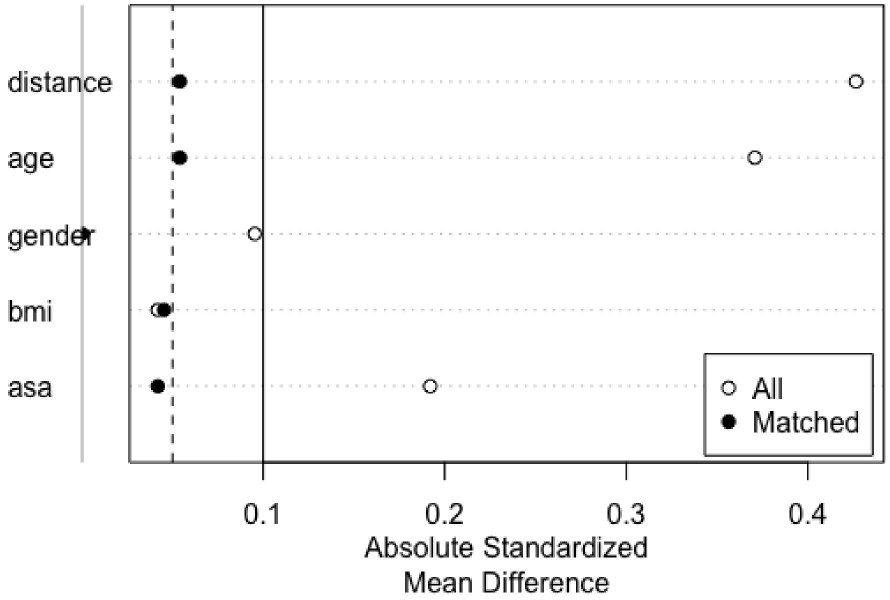

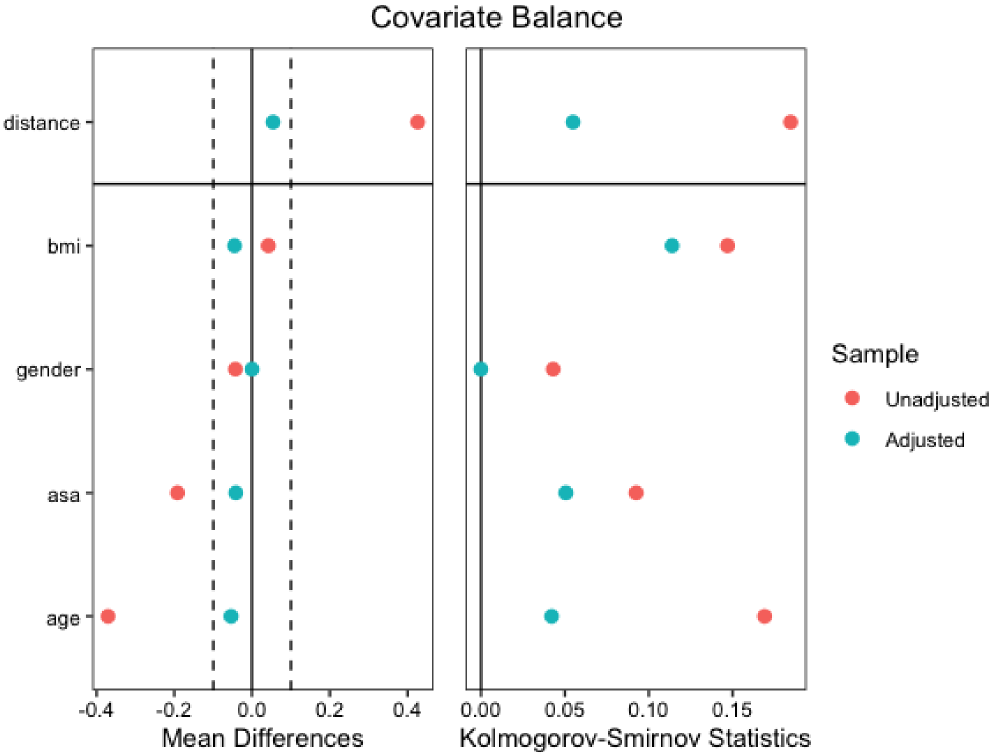

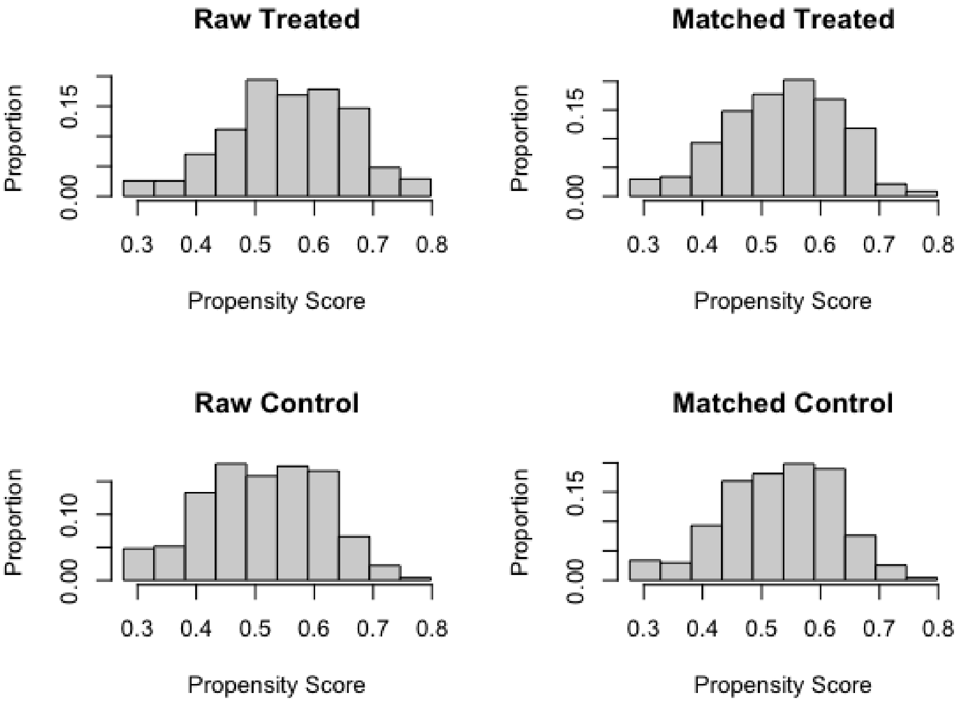
Raw Treated
Matched Treated
Raw Control
Matched Control
Proportion
Propensity Score
0.00
0.10
0.15
0.3
0.4
0.5
0.6
0.7
0.8

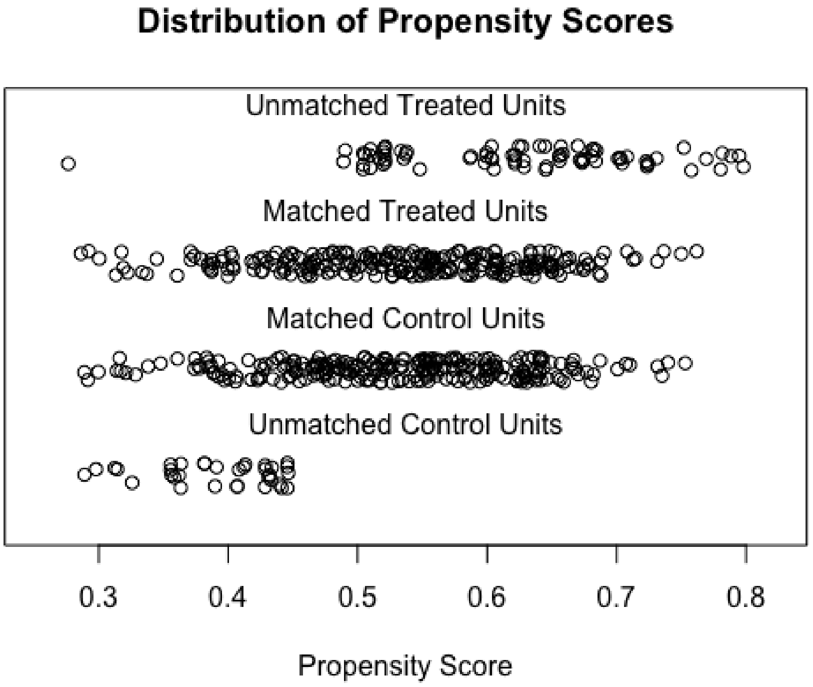
Distribution of Propensity Scores
Unmatched Treated Units
Matched Treated Units
Matched Control Units
Unmatched Control Units
0.3
0.4
0.5
0.6
0.7
0.8
Propensity Score
